# The reliability of an arabic version of the self-administered standardized chronic respiratory disease questionnaire (CRQ-SAS)

**DOI:** 10.1186/1471-2466-11-21

**Published:** 2011-04-28

**Authors:** Mohamed S Al Moamary, Hani M Tamim

**Affiliations:** 1 College of Medicine, King Saud bin Abdulaziz University for Health Sciences, Riyadh, Saudi Arabia

**Keywords:** Quality of Life, Pulmonary rehabilitation, COPD, Saudi Arabia

## Abstract

**Background:**

To produce a conceptually equivalent Arabic version to the original Self-Administered Chronic Respiratory Disease Questionnaire with standardized dyspnea domain (CRQ-SAS) and to assess its reliability.

**Methods:**

The study was carried out in two stages: stage I which was the translation of the CRQ-SAS questionnaire from the English to the Arabic language, and stage II which represented the test-retest reliability for patients receiving usual care for COPD who were not yet admitted to the pulmonary rehabilitation program.

**Results:**

Forty five patients with stable COPD were enrolled in this study. Strong test-retest reliability was found for the four domains of the CRQ-SAS, with the intra-class correlation coefficient of 0.97 for each of the domains. The association between most parameters and the four domains of CRQ-SAS were not found to be statistically significant, as measured by Pearson correlation. The number of exacerbations was negatively correlated with the dyspnea domain (correlation = -0.36, p-value = 0.02). The disease duration was negatively correlated with the domain fatigue (correlation = -0.35, p-value = 0.02). The correlation between FEV1/FVC ratio and emotion domain was -0.30 (p-value = 0.05). The mastery domain was negatively correlated with FEV1/FVC ratio with a correlation of -0.27 with borderline statistical significance (p-value = 0.07).

**Conclusion:**

The Arabic translation of the CRQ-SAS was found to be reliable to assess the quality of life among patients with COPD.

## Background

The concept of quality of life has added a new dimension to the measurement of modalities outcomes used in the management of different diseases. Health-related quality of life (HRQL) is measured by instruments specifically developed to assess the experiences of patients with diverse diseases [1]. These instruments vary from general to disease-specific such as Chronic Respiratory Disease Questionnaire (CRQ) [2,3]. The CRQ is a disease specific questionnaire utilized to assess HRQL for chronic respiratory diseases such as chronic obstructive pulmonary disease (COPD), bronchiectasis, and interstitial lung diseases. It is considered to be valid, precise, simple to use, and sensitive to change in clinical status and covers the domains of dyspnea, emotion, fatigue, and mastery [4-7]. The original CRQ was interviewer dependent (CRQ-IA) and was made available with both individualized and standardized dyspnea domain. Though CRQ-IA helps to avoid missing items and errors, it has the drawback that it should be administrated by an interviewer and may be time consuming [3,8]. This has led to the development of the CRQ - Self Administrated with standardized dyspnea domain (CRQ-SAS) [9,10]. Respondents were asked to grade their function in each item using a seven-point likert scale. The total score for each domain was divided by the number of items answered, yielding a potential score of one to seven, with higher numbers representing better function. Moreover, Schunemann et al. reported that CRQ-SAS did not impair validity and proved to have more responsiveness when compared to CRQ-IA [11].

The Arabic language is spoken by approximately 200 million people. The HRQL questionnaires are not yet widely utilized as an outcome measurement in Arabic speaking countries. This has raised the need for the availability of an Arabic version of an HRQL instrument which is socially and culturally sensitive and acceptable. Therefore, the objective of this study was to produce a conceptually equivalent Arabic version to the original CRQ-SAS with standardized dyspnea domain and to assess its reliability.

## Methods

The study was carried out at King Abdulaziz Medical City (KAMC), Riyadh, Saudi Arabia, which is a tertiary care teaching facility of approximately 1000 bed capacity. The study was carried out in two stages: stage I which was the translation of the CRQ-SAS questionnaire from the English to the Arabic language, and stage II which was the assessment of the test-retest reliability for patients receiving usual care for COPD who were not yet admitted to the pulmonary rehabilitation program (PR).

### Stage I: Translation

The process was commenced by translating the CRQ-SAS from the source language (English) to the target language (Arabic). The forward translation was conducted by two certified local translators. Both produced independent translation form the source to the target language. Apart from clarification of some medical terminology, the translators have found no difficulty during translation. After consultation with the translators, the primary author re-conciliated a first Arabic version that was considered conceptually equivalent to the source language. Another two certified translators, who were blinded to the source documents, carried out the back translation from Arabic to English. Both produced independent translation from the target language (first version of CRQ-SAS) to the source language. The primary author compared the two translations with the source CRQ-SAS. The back translation version and the original source document were reviewed by the owner of CRQ-SAS. Based on the received comments, a final Arabic version was produced. Finally, a pilot test was carried out on 5 patients to ensure that the final draft is clear and understandable. Based on this testing, we have finalized the Arabic version of the CRQ-SAS.

### Stage II: Test - retest reliability

The test-retest reliability was conducted at the out-patient pulmonary clinic and the PR program at the KAMC-Riyadh. It was carried out by recruiting a total of 45 patients attending either of the above mentioned departments. The inclusion criteria were: age ≤ 75 years, clinical diagnosis of COPD in a stable condition for at least 4 weeks, the ratio of forced expiratory volume in one second over forced expiratory volume (FEV_1_/FVC ratio) ≤ 70% and FEV_1 _≤ 70%, and free of significant handicapping disease. After signing the informed consent form, patients were interviewed and physically examined as part of their routine management. The Arabic version of the CRQ-SAS was initially self-administrated to the patients. The re-test session was arranged after 3-5 weeks taking in consideration that they were in a stable clinical condition. Moreover, patients underwent different standard tests that included: measurement of FEV_1_, FVC, FEV_1_/FVC ratio, residual volume (RV), total lung capacity (TLC), arterial saturation by pulse oximeter, and the 6-minute walking test (6MWT) as per American Thoracic Society criteria [12,13]. Pulmonary function test would normally be done within two weeks from the 6MWD and CRQ-SAS to avoid exhausting patients. This study received the approval of the institute review board of King Abdullah International Centre for Medical Research (KAIMRC), the research arm of our health organization.

### Statistical analysis

The statistical analyses carried for stage II of this study are presented in this subsection. Data were entered into the Statistical Package for Social Sciences (SPSS) version 16, which was used for the data management and analyses. Continuous variables were summarized by calculating the mean and its standard deviation, whereas categorical variables were summarized by the number and percent. Pearson correlation coefficients were calculated for the different measures of the CRQ-SAS and the different baseline characteristics. Reliability analysis was carried out by calculating the intraclass correlation coefficient for the test-retest reliability of the Arabic version of the CRQ-SAS. A value of 0.7 was considered acceptable both for Crohnbach's alpha and the intraclass correlation coefficient.

## Results

Forty five patients with stable COPD were enrolled in this study. Their average age was 63.3 ± 8.8 years and ranging between 44-75 years. There were 19 female patients (42.2%) and 26 male patients (57.8%). The average disease duration was 13.9 years ± 7.1 (range from 4 to 30). The smoking history reflected as the pack-year was found to be 39.7 ± 35.8 (range from 0 to 150). The average number of exacerbations over the past 12 months was found to be 3.8 ± 2.5 (range from 1 to 10), whereas the number of admissions was 1.2 ± 1.4 (range from 0 to 5). The average body mass index for the subjects was 32.6 ± 9.3 (range from 18.4 to 52.4). The FEV_1_, FVC, and FEV_1_/FVC ratio were 58.4% ± 15.3 (range from 25 to 78), 70.1% ± 12.7 (range from 32 to 85), and 61.7 ± 8.8 (range from 38 to 78), respectively. The total lung capacity was 90.4% ± 14.8 (range from 71 to 145). 6MWD was 226.9 meter ± 99.2 (range from 50 to 400). Finally, pertaining to the medications, it was found that 41 patients (91.1%) used an inhaled corticosteroid agent, 42 (95.6%) used a long acting beta agonist agent, and 30 (66.7%) used tiotropium bromide.

**Figure 1:**
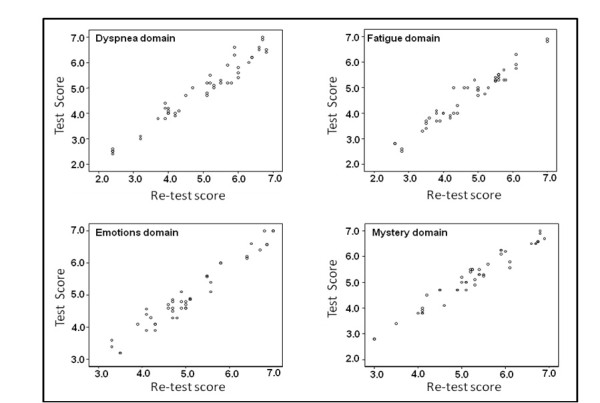
**A scatter plot for the responses of the subjects on the 4 domains of the chronic respiratory disease questionnaire**.

The time difference between test-retest CRQ administrations was 4 ± 0.8 weeks. Table 1 presents the intraclass correlation coefficients, as well as the means and standard deviations, between the baseline and follow-up as an assessment of the test-retest reliability. Strong test-retest reliability was found for the four domains of the CRQ-SAS, with the correlation coefficient of 0.97 for each of the domains. Figure 1 presents the scatter plot for the Reponses of the subjects on the 4 domains of the CRQ-SAS, which indicates high correlation.

**Table 1:** Intraclass correlation coefficients between baseline and follow-up as an assessment of the test-retest reliability of the 45 consecutive patients with COPD enrolled in the study (mean ± standard deviation, and range)

Domain	Test Score Mean ± standard deviation (range)	Re-test Score Mean ± standard deviation (range)	Intraclass correlation
Dyspnea	4.9 ± 1.3 (2.4 - 7.0)	5.0 ± 1.3 (2.4 - 6.8)	0.97

Fatigue	4.6 ± 1.1 (2.5 - 6.9)	4.7 ± 1.1 (2.6 - 7.0)	0.97

Emotions	5.0 ± 1.0 (3.2 - 7.0)	5.1 ± 1.0 (3.3 - 7.0)	0.97

Mystery	5.2 ± 1.1 (2.8 - 7.0)	5.3 ± 1.0 (3.0 - 6.9)	0.97

Table 2 presents the correlations between selected parameters and the four domains of CRQ-SAS. Most of the correlations calculated were not found to be statistically significant. On the other hand, number of exacerbations over the past 12 months was found to be negatively correlated with the dyspnea domain (correlation = -0.36, p-value = 0.02). The disease duration was also found to be negatively correlated with the domain fatigue (correlation = -0.35, p-value = 0.02). The correlation between FEV_1_/FVC ratio and emotion domain was -0.30 (p-value = 0.05). Finally, the mastery domain was also negatively correlated with FEV_1_/FVC ratio with a correlation of -0.27, which was found to be of borderline statistical significance (p-value = 0.07). Both the FEV_1 _and the 6MWD did not show significant correlation with any of the domains.

**Table 2:** Correlations between the initial score of the four domains of CRQ and selected parameters

	Dyspnea*	Fatigue*	Emotion*	Mastery*
Age	0.02 (0.88)	0.07 (0.65)	0.05 (0.75)	0.11 (0.49)

Disease duration	-0.23 (0.13)	-0.35 (0.02)	-0.25 (0.10)	-0.09 (0.58)

Number of exacerbations over twelve months	-0.36 (0.02)	-0.02 (0.88)	-0.002 (0.99)	-0.07 (0.66)

Number of admissions over twelve months	-0.18 (0.23)	-0.06 (0.68)	-0.03 (0.85)	-0.20 (0.18)

Smoking history (Pack-year)	-0.04 (0.77)	-0.17 (0.26)	-0.13 (0.39)	-0.20 (0.19)

The percentage of Forced Expiratory volume in one second (FEV_1_)	0.08 (0.59)	0.18 (0.25)	-0.07 (0.64)	0.02 (0.91)

FEV1/FVC ratio	-0.02 (0.91)	-0.20 (0.19)	-0.30 (0.05)	-0.27 (0.07)

Six minutes walk distance (meter)	-0.02 (0.92)	0.06 (0.69)	0.05 (0.76)	-0.05 (0.76)

Body mass index	-0.12 (0.45)	-0.26 (0.08)	-0.10 (0.50)	0.0 (1.00)

*correlation (p value)

## Discussion

The present study confirms that the Arabic version of the CRQ-SAS performed well and was reliable with a strong interclass co-relation. Moreover, to the author's knowledge, the current study is the first that ever assessed the quality of life of patients with COPD from Saudi Arabia. This concept of HRQL was not commonly practiced in the Middle East as reflected by the scanty data that came from this region. Its importance rose from the fact that physiological measures may not sufficiently assess functional outcomes of interventions implemented for COPD where HRQL instruments can serve this purpose [1,3].

The instruments that measure HRQL for COPD could be either disease specific or general in nature. When compared to general tools, the disease specific instruments are more responsive and may be more valid to both clinician and patients [1,7]. An effective instrument should be valid, interpretable, responsive, and reliable [14,15]. The CRQ which was developed in 1987 is one of the most commonly used tools to assess HRQL in patients with COPD that fulfill the aforementioned criteria [7,13]. Though the original CRQ was simple to use, it was time consuming as it is interviewer dependent with individualized dyspnea domain. In one study, the time required to complete the CRQ-SAS was reduced to half the time required for the CRQ-IA [16]. This is partly related to the standardization of the items related to the dyspnea domain. The CRQ-SAS is constituted of 20 items that cover four domains: dyspnoea with 5 questions, fatigue with 4 questions, emotion with 7 questions, and mastery (Feeling of control over the disease) with 4 questions. The brevity of the CRQ-SAS with standardized dyspnea domain was considered to be an attractive alternative as it is also valid, reliable, and responsive [10,12].

A valid translation is essential to achieve a HRQL instrument that functions well in a different environment from its original setting. This carries more importance in questionnaires that assess a wide spectrum of domains like CRQ. Arabic Language is spoken in 21 countries which necessitate paying attention to the slight differences related to variations in the accents of various regions. Our translation was adapted to the region of the six countries of the Gulf Cooperation Council (Bahrain, Kuwait, Oman, Qatar, Saudi Arabia, and United Arab Emirates). The lengthy process of translating CRQ-SAS to Arabic has resulted in the adaptation of a conceptually equivalent Arabic version to the original CRQ-SAS. The achieved test-retest reliability in our study reflects stability over time while the strong internal consistency reflects stability across the items of each domain. Similar finding were achieved by the translation of CRQ to other languages like Dutch, Spanish, French-Canadian, and Korean [16-19]. When compared to other studies, patients with COPD from Saudi Arabia were affected by their disease as reflected by measuring their quality of life by CRQ-SAS (Table 3)

**Table 3:** Comparison of the different studies in the literature that assessed the health related quality by CRQ-SAS and the current one

Study	Year	Country	FEV_1_	Dyspnea	Fatigue	Emotions	Mastery
Williams et al [10]	2001	UK	1.13 L	2.4	3.3	4.4	4.2

Puhan et al [12]	2004	Multinational	45.1%	3.6	3.8	4.1	4.25

Schunemann et al [13]	2005	Canada/USA	45.4%	4.1	3.9	4.7	4.6

Oh et al [23]	2009	Korea	52.3%	4.4	4.0	4.6	5.2

Al-Moamary et al (current study)	2009	Saudi Arabia	58.4%	4.9	4.6	5.0	5.2

Similar to the findings in the literature, our study showed lower correlations between CRQ domains with the nature of COPD and pulmonary function parameters [3,18,20]. In our study, dyspnea was highly correlated to the number of execrations which reflects a disease that either advanced or poorly controlled [21,22]. Dyspnea was not linked with pulmonary function, a finding supported by other studies [17,22]. Fatigue domain was highly correlated with disease duration, while both emotion and mastery domains were correlated with a more severe disease as reflected by FEV1/FVC ratio. Guell et al found higher correlation between the last two domains and pulmonary function [17]. The domains of emotion, fatigue, and mastery were associated with anxiety and depression, findings that are commonly observed in patient with either advanced disease or longer disease duration. Despite documented correlation of CRQ-SAS with the 6MWD, our finding did not show such correlation [10,12,13,23]. A finding that can be explained with higher FEV_1 _in our study when compared to other studies shown in table 3 and the need to a larger study sample to verify the lack of correlations.

Finally, we would like to mention some limitations of the current study. Due to unavailability of a gold standard test to assess HRQL for patients with COPD by a valid linguistic translation of similar instruments, this study was limited to the reliability of the CRQ-SAS and not extended to assess its validity. Though we belief that the lack of priori hypotheses can be claimed to conclude sufficient validity. Moreover, responsive of the Arabic version of CRQ-SAS to change was not planned in the design of the study and merit further studies to assess this aspect.

## Conclusion

The Arabic translation of the CRQ-SAS was found to be reliable among patients with COPD. It can also perform well to assess their HRQL in a manner similar to the original CRQ-SAS.

## Competing interests

The authors declare that they have no competing interests.

## Authors' contributions

MA-M is the principle investigator who contributed in study design, getting approval, patients recruitment, data collection and analysis, writing the paper, and submission. HT has contributed in study design, data analysis, and writing the paper.

## Pre-publication history

The pre-publication history for this paper can be accessed here:

http://www.biomedcentral.com/1471-2466/11/21/prepub
